# The relevance of BRAF G469A mutation in determining the response to therapy in metastatic melanoma

**DOI:** 10.1186/1479-5876-13-S1-P3

**Published:** 2015-01-15

**Authors:** Letizia Porcelli, Gabriella Guida, Tiziana Cocco, Anna E Quatrale, Rosa M Iacobazzi, Diana A Stolfa, Imma Maida, Anna Ferretta, Claudia Grieco, Stefania Guida, Sabino Strippoli, Stefania Tommasi, Michele Guida, Amalia Azzariti

**Affiliations:** 1 Clinical Experimental Oncology Laboratory, Istituto Tumori Giovanni Paolo II, Bari, Italy; 2 Dept. of Basic Medical Sciences, Neurosciences and Sense Organs, University of Bari 'A. Moro', 70124 Bari, Italy; 3 Department of Medical Biochemistry, Biology & Physics, University of Bari 'A. Moro', 70124 Bari, Italy; 4 Medical Oncology Department, Istituto Tumori Giovanni Paolo II, Bari, Italy

## Background

BRAF G469A is a missense mutation within exon 11 of the BRAF gene resulting in a constitutively active state of the enzyme responsible also for the protein localization to the mitochondria. The BRAF G469A mutation, frequently recovers in lung cancer, is rare in melanoma and uncertain is its association with a more aggressive disease. BRAF G469A mutation is frequently associated to MAP kinase cascade signaling activation, however no evidence currently exists about its role in determining sensitivity/resistance to BRAF inhibitors vemurafenib or dabrafenib. In our Institute, a patient with metastatic melanoma (MM) was treated with fotemustine, however the disease progressed. From patient biopsy, a new metastatic melanoma cell line has been established and named Mo-1. Here, we investigated the sensitivity of Mo-1 cells to vemurafenib and abraxane, both already approved for the treatment of melanoma carrying BRAF V600 mutations, comparing it with that found in MM cells wild type for BRAF or mutated in V600. Furthermore, a biomarker for the response to abraxane is hypothesized.

## Materials and methods

We tested the sensitivity of Mo-1 (BRAF G469A), HBL and LND-1 (BRAF wild type), MBA72 and Hmel-1 (BRAF V600) to abraxane and vemurafenib by MTT cytotoxicity assay. In addition, cellular effectors were investigated by ELISA kits, western blotting and flow cytometry.

## Results

The exposure to vemurafenib leads to a progressive inhibition of proliferation however, the concentrations utilized are high and similar to those utilized in MM models constitutively resistant to BRAF inhibitors. Thus, the BRAF mutation G469A confers resistance to vemurafenib also confirmed by the partial response obtained when the patients was treated with the drug. The cellular effector responsible for the resistance might be the activation of Erk1/2.

The binding of BRAF G469A to the outer mitochondrial membrane suggested to evaluate the mitochondria functionality in Mo-1 cells. An high production of ATP and the release of lactate were evident.

Finally, abraxane, which induces mitochondria-mediated apoptosis, strongly reduced proliferation in Mo-1 cells. Possible biomarker responsible for this response is the very low expression level of PMEL17, transcriptionally regulated by MITF, which is known to be negatively involved in determining the activity of taxanes.

## Conclusion

Thus, the mutation BRAF G469A in patients with MM should be related to a weak effectiveness of therapy with BRAF inhibitors and a promising therapeutic approach may be with abraxane.

